# Microvascular insulin resistance with enhanced muscle glucose disposal in CD36 deficiency

**DOI:** 10.1007/s00125-024-06292-4

**Published:** 2024-11-06

**Authors:** Cyndya A. Shibao, Vivek S. Peche, Terri A. Pietka, Dmitri Samovski, Ian M. Williams, Naji N. Abumrad, Eric R. Gamazon, Ira J. Goldberg, David H. Wasserman, Nada A. Abumrad

**Affiliations:** 1 Department of Medicine, Division of Clinical Pharmacology, Vanderbilt University Medical Center, Nashville, TN USA; 2 Department of Medicine, Division of Nutritional Sciences and Obesity Research, Washington University School of Medicine, St Louis, MO USA; 3 Department of Molecular Physiology and Biophysics, Vanderbilt University Medical Center, Nashville, TN USA; 4 Department of Surgery, Vanderbilt University Medical Center, Nashville, TN USA; 5 Department of Medicine, Division of Genetic Medicine, Vanderbilt University, Nashville, TN USA; 6 Department of Medicine, Division of Endocrinology, Diabetes and Metabolism, New York University Grossman School of Medicine, New York, NY USA; 7 Department of Cell Biology & Physiology, Washington University School of Medicine, St Louis, MO USA

**Keywords:** African Americans, Caveolin, Endothelial function, Nitric oxide, rs3211938

## Abstract

**Aims/hypothesis:**

Microvascular dysfunction contributes to insulin resistance. CD36, a fatty acid transporter and modulator of insulin signalling, is abundant in microvascular endothelial cells. Humans carrying the minor allele (G) of *CD36* coding variant rs3211938 have 50% reduced CD36 expression and show endothelial dysfunction. We aimed to determine whether G allele carriers have microvascular resistance to insulin and, if so, how this affects glucose disposal.

**Methods:**

Our multi-disciplinary approach included hyperinsulinaemic–euglycaemic clamps in *Cd36*^−/−^ and wild-type mice, and in individuals with 50% CD36 deficiency, together with control counterparts, in addition to primary human-derived microvascular endothelial cells with/without CD36 depletion.

**Results:**

Insulin clamps showed that *Cd36*^−/−^ mice have enhanced insulin-stimulated glucose disposal but reduced vascular compliance and capillary perfusion. Intravital microscopy of the gastrocnemius showed unaltered transcapillary insulin flux. CD36-deficient humans had better insulin-stimulated glucose disposal but insulin-unresponsive microvascular blood volume (MBV). Human microvascular cells depleted of CD36 showed impaired insulin activation of Akt, endothelial NO synthase and NO generation. Thus, in CD36 deficiency, microvascular insulin resistance paradoxically associated with enhanced insulin sensitivity of glucose disposal.

**Conclusions/interpretation:**

CD36 deficiency was previously shown to reduce muscle/heart fatty acid uptake, whereas here we showed that it reduced vascular compliance and the ability of insulin to increase MBV for optimising glucose and oxygen delivery. The muscle and heart respond to these energy challenges by transcriptional remodelling priming the tissue for insulin-stimulated glycolytic flux. Reduced oxygen delivery activating hypoxia-induced factors, endothelial release of growth factors or small intracellular vesicles might mediate this adaptation. Targeting NO bioavailability in CD36 deficiency could benefit the microvasculature and muscle/heart metabolism.

**Trial registration:**

Clinicaltrials.gov NCT03012386

**Data availability:**

The RNAseq data generated in this study have been deposited in the NCBI Gene Expression Omnibus (www.ncbi.nlm.nih.gov/geo/) under accession code GSE235988 (https://www.ncbi.nlm.nih.gov/geo/query/acc.cgi?acc=GSE235988).

**Graphical Abstract:**

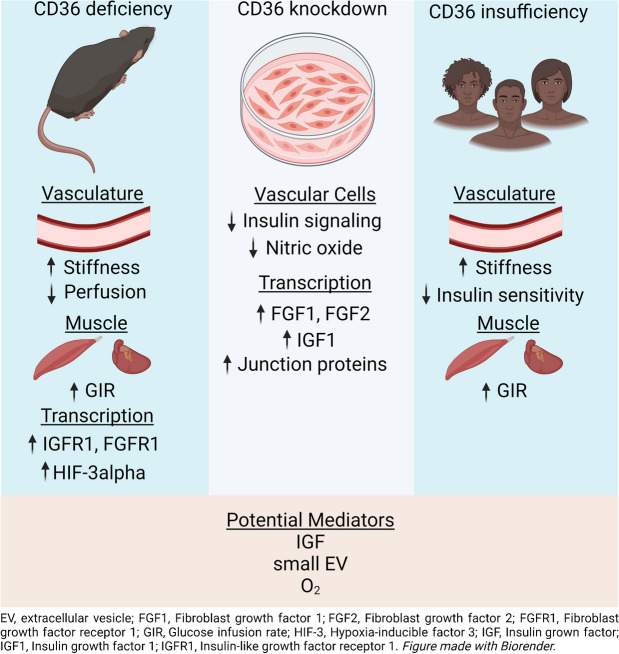

**Supplementary Information:**

The online version contains peer-reviewed but unedited supplementary material available at 10.1007/s00125-024-06292-4.


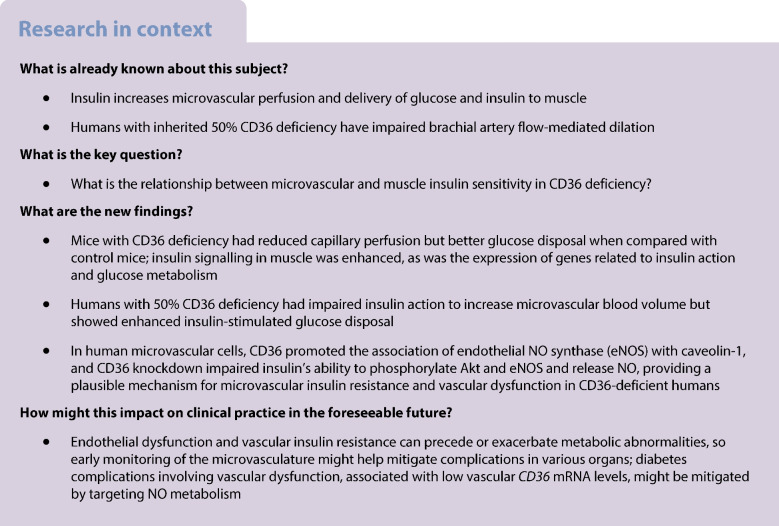


## Introduction

The gene encoding for membrane fatty acid transporter CD36 was identified as an insulin resistance gene when *CD36* variants were found in insulin-resistant spontaneously hypertensive rats (SHR) and the SHR’s metabolism improved with CD36 expression [1, 2]. In humans, a *CD36* SNP (rs1527479) associates with insulin resistance and type 2 diabetes [3] and a mutation (rs56381858) associates with autosomal-dominant type 2 diabetes [4]. Only a few studies have examined insulin-stimulated glucose disposal in partial or total CD36-deficient individuals, yielding conflicting results [5–7].

Complete CD36 deficiency has higher incidence (0.3–11%) in African and Asian populations [8, 9]. Coding SNP rs3211938 (G/T) causes total CD36 deficiency in African Americans and the minor G allele (20–25% frequency) reduces CD36 protein levels by 50% [10, 11]. Common SNPs in white individuals reduce CD36 levels [12]. In mice, CD36 deficiency (*Cd36*^−/−^) enhances glucose disposal [13] but is not recapitulated by muscle *Cd36* deletion [14]. In contrast, endothelial-cell *CD36* deletion mimics the better glucose disposal of *Cd36*^−/−^ mice [15], suggesting that muscle glucose uptake is critically influenced by endothelial-cell CD36.

The endothelium, notably the microvasculature, controls nutrient uptake, muscle insulin access [16, 17] and glucose disposal [18, 19]. Obesity-associated endothelial dysfunction delays the action of insulin in increasing microvascular blood volume (MBV) and reduces systemic glucose uptake [20]. We reported that 50%-CD36-deficient humans carrying the G allele of rs3211938 have reduced flow-mediated dilation of the brachial artery and low cGMP, suggesting diminished NO bioavailability [10]. Here, we tested the hypothesis that G allele carriers have impaired insulin-induced increase of MBV and that this might influence muscle glucose disposal. Using metabolic insulin clamps, we assessed insulin-sensitive glucose disposal in 50%-CD36-deficient humans and in *Cd36*^−/−^ mice, and examined microvascular function, transendothelial insulin flux and glucose uptake.

## Methods

### Mice

Age- (3 ± 1 months) and sex-matched C57BL/6 wild-type (WT) and *Cd36*^−/−^ mice, generated as previously described [21], were used in all studies except for the insulin clamp in which male mice were used. Studies were approved by Institutional Animal Care and Use Committees (IACUC) of Washington University and Vanderbilt University. For the animal studies, no randomisation was used. Sets of animals were all studied at the same time.

#### Vessel compliance

Ascending aortas and left carotid arteries dissected from euthanised WT and *Cd36*^−/−^ mice [22] were mounted in saline (154 mmol/l NaCl) on a pressure arteriograph and then pressurised and stretched to in vivo length three times before data capture. Vessel diameter recordings used a transillumination microscope with camera and computerised measurements (Myoview, Danish Myotechnology). Intravascular pressure was increased in 25 mmHg steps (from 0 mmHg to 175 mmHg) and the outer diameter of vessels was measured.

#### Muscle insulin sensitivity

Mice were fasted for 5 h and then given an i.p. injection of insulin (0.75 U/kg). Tissues were harvested 15 min later and then frozen in liquid nitrogen at −80°C. Tissues were lysed in Cell Lysis Buffer (9803) from Cell Signalling, USA. Western blots were performed as described [14] using anti-mouse CD36 1:2000 from R&D Systems USA (AF1955) and anti-pS473-Akt 1:1000 (4060), anti-pT308-Akt 1:1000 (13038) and total Akt 1:1000 (4691), all from Cell Signalling, USA. Dilutions were in 5% bovine serum albumin, 1 × TBS and 0.1% Tween 20.

#### Transcapillary insulin flux

Intravital microscopy of Alexa Fluor 647-conjugated insulin (INS-647) was used to visualise transcapillary insulin flux in the exposed gastrocnemius of anaesthetised (ketamine/xylazine/acepromazine, 7.9/1.6/0.2 mg/kg) *Cd36*^−/−^ and WT mice as described previously [23].

Blood vessels were visualised as follows: 50 μg of 2 MDa Rhodamine–dextran infusion; Zeiss Filter Set 43 HE; excitation 550/25 nm, fluorescence tomography (FT) 570 nm, emission bandpass (BP) 605/70. The field of view, selected from a capillary bed stemming from the external sural artery, had sufficient capillaries, no nearby large vessels or immediately adjacent capillaries. After imaging region selection, a background image (t=0) was acquired, probe (Rhodamine–dextran and INS-647) was injected through the venous catheter and images were acquired every minute (1–10 min) and at 12.5 min and 15 min post probe injection. See [23] and electronic supplementary material (ESM) Methods (Mouse studies) for further details.

#### Capillary perfusion area in kidneys of WT and *Cd36*^−/−^ mice

Non-invasive arterial spin labelling (ASL) MRI was used for quantitative measure of microvascular tissue perfusion in WT and *Cd36*^−/−^ mice. Data (*n*=5/group) were collected at baseline (light anaesthesia; 1% isoflurane in 100% O_2_). See ESM Methods (Mouse studies) for further details.

#### Capillary density

Heart capillaries of WT and *Cd36*^−/−^ mice were immunostained for junctional adhesion molecule 3. See ESM Methods (Mouse studies) for details.

#### Hyperinsulinaemic–euglycaemic insulin clamp

Catheters were implanted under isoflurane anaesthesia in the carotid artery for sampling and jugular vein for infusion 1 week before performing hyperinsulinaemic–euglycaemic clamps (HIECs). During the clamps, the mice (fasted for 5 h) were not restrained or handled [24]. [3-^3^H]Glucose was primed and continuously infused from t=−90 min to t=0 min (1480 Bq [0.04 µCi]/min) and the clamp was initiated at t=0 min, with continuous insulin infusion (4 mU kg^−1^ min^−1^) and variable glucose infusion rate (GIR), both maintained until t=155 min. The infused glucose had 2220 Bq (0.06 µCi)/µl [3-^3^H]-glucose to minimise changes in plasma [3-^3^H]-glucose specific activity. Arterial glucose was measured every 10 min and the GIR was adjusted to maintain euglycaemia. Erythrocytes were infused to compensate for withdrawn blood. [3-^3^H]glucose kinetics were determined at −15min and −5min (basal period) and every 10 min between 80 min and 120 min to assess whole-body glucose appearance rate (*R*_a_), whole-body glucose disappearance rate (*R*_d_) and endogenous glucose production rate (endo*R*_a_). To determine the glucose metabolic index (tissue-specific glucose uptake rate, *R*_g_), 481,000 Bq (13 µCi) of ^14^C-labelled 2-deoxyglucose ([^14^C]2DG) was given intravenously at 120 min and blood collected at 122, 125, 135, 145 and 155 min. to assess [^14^C]2DG disappearance. [^14^C]2DG is transported and phosphorylated like glucose but is not metabolised further so excised tissues were analysed for [^14^C]2DG phosphate. The metabolic index was calculated as follows: tissue [^14^C]2DG phosphate divided by AUC for plasma [^14^C]2DG, then multiplied by plasma glucose. Plasma [3-^3^H]glucose and [^14^C]2DG and tissue [^14^C]2DG phosphate were measured as described in [25], and detailed online (Vanderbilt Mouse Metabolic Phenotyping Center; www.vmmpc.org).

#### Microarray

Analysis of gene expression in heart muscle from WT and *Cd36*^−/−^ mice fasted for 5 h (*n*=4/group) used Mouse Genome Oligo Microarray (Agilent) and Washington University Functional Genomics Core.

### Humans

#### Participants

Healthy unrelated African Americans self-identified as men or women aged 18–50 years were genotyped for *CD36* coding SNP rs3211938 (G/T). This SNP results in a truncated protein that is degraded; 50% CD36 protein deficiency is observed in G allele carriers (incidence 20–25%) and 100% deficiency in homozygous (G/G) [10, 11]. We excluded individuals with BMI>40 kg/m^2^, type 1 diabetes, type 2 diabetes, hypertension, impaired renal function and impaired liver function and those using nitrate or glucocorticoid. Genotyping for rs3211938 used a predesigned TaqMan SNP genotyping assay (Applied Biosystems) on a 7500 Fast (Applied Biosystems) instrument, as described previously [10, 11]. G allele carriers and non-carriers underwent a screening visit for medical history, physical examination and laboratory analyses (blood cell count, metabolic panel, pregnancy test).

All participants provided informed consent and underwent two visits at the Vanderbilt Clinical Research Center (CRC) at 08:00 hours in a quiet, temperature-controlled room (22–23°C). Participants were asked to abstain from exercise or alcohol for ≥24 h before the study and were reminded to fast the night before. For the human studies, masking or blinding was not possible as the experimenters received an insulin clamp with/without intralipid. All studies were approved by Vanderbilt Institutional Review Board and adhered to the Declaration of Helsinki’s principles and Title 45 of US Code of Federal Regulations (Part 46, Protection of Human Subjects).

#### HIEC

During each visit, HIECs were performed. A linear-array transducer connected to an ultrasound was placed on the brachioradialis muscle of the arm not used for infusion for assessment of microvascular circulation with contrast-enhanced ultrasonography (CEU).

On day 1 (saline day), participants received 0.9% saline infusion (45 ml/h) for 6 h and, during the last 3 h, an HIEC clamp. Insulin was infused for 5 min at 80 mU m^−2^ min^−1^ followed by 40 mU m^−2^ min^−1^ for the study remainder. Plasma glucose samples were obtained every 5 min throughout and 20% glucose was infused at a variable rate to maintain plasma glucose at 5–5.27 mmol/l. See ESM Methods (Human studies) for more details on measurements of plasma glucose and serum insulin.

On day 2, ~4 weeks later, participants returned and instead of saline received a 6 h i.v. infusion (45 ml/h) of 20% Intralipid (Baxter Healthcare) and heparin (200 U priming dose, then 200 U/h) to activate endothelial fatty acid lipolysis from triacylglycerols. HIECs were conducted over the last 3 h of the study.

At both visits, CEU measurements were obtained at baseline and 3 h after infusion initiation (saline or Intralipid/heparin) during the insulin clamp. Intermittent BP and ECG were measured. Vanderbilt’s Investigational Pharmacy handled drug preparation, storage and dispensing logs.

Blood samples obtained during the hyperinsulinaemic–euglycaemic clamp were not arterialised since the sampling hand was not heated.

#### Assessment of MBV, microvascular blood flow and overall perfusion

MBV change after insulin was measured as described [26] by contrast-enhanced ultrasound (linear-array transducer connected to an ultrasound, L9–3 mm, iU22; Phillips). Real-time imaging used low (0.08) and high (1.2) mechanical index. Contrast microbubbles (Definity, Bristol-Myers Squibb) were activated by vial mixer (Lantheus Medical Imaging) at 4500 oscillations/min for 45 s. Microbubbles (1.5 ml suspension) diluted to 20 ml with sterile saline were intravenously infused (1.5 ml/min,10 min) using a rotating syringe pump. At steady state (~4 min), microbubbles were destroyed by the 1.2 index, and video recording started. Switching to the low index (0.08) allowed real-time recording of vascular microbubble replenishment. All MBV data were from four 45 s imaging periods. The frame obtained during the first 0.5 s after microbubble destruction was used for subtracting tissue background and signal from microbubbles in larger fast-flowing blood vessels to obtain MBV, velocity (1/s) of microvascular blood flow (MBF), and overall perfusion (MBV×MBF). Data analysis used QLAB software (https://qlab.app). Local temperature was measured with a laser Non-Contact Infrared Skin Thermometer (Globe Scientific, Denmark)

### Human microvascular endothelial cells

Human-derived primary dermal microvascular endothelial cells (hMECs) (Lonza Bioscience), cultured in EGM-2 MV endothelial media (Lonza), were treated with anti-CD36 or control siRNA (ThermoFisher Scientific) and 72 h later were used for RNAseq [27]. See ESM Methods (hMECs) for further details.

Cells were serum starved for 4 h before the addition of human insulin (100 nmol/l, Sigma) and lysis (20 min) in ice-cold RIPA buffer (9806 Cell Signaling). Proteins (30 µg) from cleared lysates (10,000 g, 10 min) were separated on 4%–20% gradient gels (ThermoFisher) and transferred to polyvinylidene membranes (Immobilon Fl; Millipore). Membranes were blocked (1 × TBS, 0.25% fish gelatine, 0.01% sodium azide, 0.05% Tween-20) and primary antibodies were added overnight at 4°C (anti-human CD36 [R&D Systems], β-actin [Santa Cruz, sc-47778] and anti-pS473-Akt, total Akt, pS1177-endothelial NO synthase [eNOS] [9571] and total eNOS [32027, Cell Signaling]). All antibodies were added at 1:1000 dilution. Membranes were treated at room temperature for 1 h with infrared dye-labelled secondary antibodies (LI-COR Biosciences) and imaged (LI-COR Odyssey).

For immunoprecipitation (IP) serum-starved (4 h) hMECs were treated with insulin (100 nmol/l, 10 min, 37°C), washed and scraped into 1 ml IP buffer (0.1 mol/l NaCl, 0.3 mol/l Sucrose, 30 mmol/l MgCl2, 10 mmol/l PIPES, 0.5 mmol/l EDTA, 0.1% Nonidet P-40, protease and phosphatase inhibitors, and 0.5 mmol/l pervanadate) [27]. Protein aliquots (50 µg) were incubated for 5 h with caveolin-1 (Cav-1) antibody (Cell Signaling, 3238) coupled to protein G magnetic beads (Dynabeads; ThermoFisher) and rabbit IgG-coupled beads were used as a control. Immune complexes were separated magnetically and boiled in 50 µl 2 × SDS Sample buffer prior to SDS-PAGE (4–12%). Further details on hMEC handling, western blot analysis and IP are available in [27].

#### NO measurement

The Nitrate/Nitrite Colorimetric Assay (Cayman Chemicals, 780001) was used. Human insulin (Sigma, I9278) was added (100 nmol/l, 10 min) to cells that had been serum starved for 4 h and media were collected for NO measurement as per the manufacturer’s protocol.

#### Expression of microvascular junction protein

CD36-depleted and control hMECs (*n*=5 preparations per condition) were used. See ESM Methods (Human microvascular cells) for further details and ESM Table 1 for primers used.

### PrediXcan analysis

The genetically determined component of CD36 expression was estimated from gene expression imputation trained with reference transcriptome data (*N*=44 tissues, 449 donors, version 6p) from the Genotype-Tissue Expression (GTEx) Consortium [28] as described previously [29].

### Biostatistics

Data are presented as means ± SD. Data were log-transformed prior to statistical testing when not normally distributed by the Shapiro–Wilks test. Summary data were analysed by a parametric, two-tailed Student’s *t* test. *p* values of <0.05 were considered significant. Analyses used Prism 10.0 (GraphPad Software; https://www.graphpad.com/).

## Results

### *Cd36*^−/−^ mice display enhanced glucose disposal and muscle insulin signalling

**Fig. 1:**
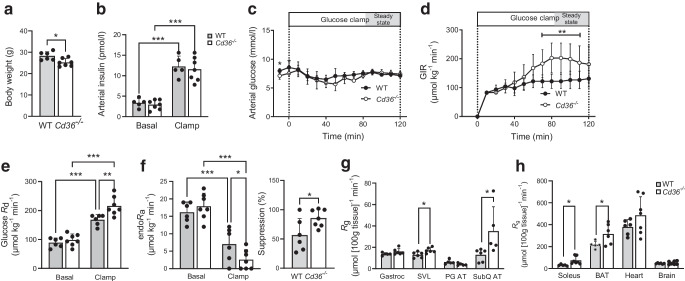
CD36 deletion in mice improves insulin sensitivity and the *R*_g_. Hyperinsulinaemic–euglycaemic clamps were performed in awake, non-restrained WT and *Cd36*^−/−^ mice. (**a**) Body weight. (**b**) Fasting and clamp insulin was similar in both genotypes. (**c**) Fasting glucose was reduced in *Cd36*^−/−^ but blood glucose levels were similar in WT and *Cd36*^−/−^ mice during the clamp. (**d**) A higher GIR was needed to maintain euglycaemia in *Cd36*^−/−^ mice compared with WT mice. (**e**) The *R*_d_ was higher in *Cd36*^−/−^ mice during the clamp. (**f**) Insulin more effectively suppressed endo*R*_a_ in *Cd36*^−/−^ mice. (**g**, **h**) Glucose metabolic index in gastrocnemius, superficial vastus muscle, perigonadal adipose tissue and subcutaneous adipose tissue (**g**) and in soleus, brown adipose tissue, heart and brain (**h**) in *Cd36*^*−/−*^ mice compared with WT mice. Gastrocnemius, peri-gonadal adipose tissue and heart *R*_g_ were elevated but differences did not reach significance. As expected, brain *R*_g_ was equivalent in WT and *Cd36*^−/−^ mice. Data are shown as means ± SD. *n*=6 control mice and *n*=7 *Cd36*^−/−^ mice. *p<0.05, **p<0.01, ***p<0.001. BAT, brown adipose tissue; Gastroc, gastrocnemius; PG AT, perigonadal adipose tissue; SVL, superficial vastus muscle; SubQ AT, subcutaneous adipose tissue

*Cd36*^−/−^ mice had 8% lower body weight than WT mice (Fig. 1a); lean body mass is generally similar [30]. Plasma insulin is similar but *Cd36*^−/−^ mice have lower arterial glucose, as reported in earlier studies [13, 15]. Insulin clamps were performed in 5 h-fasted mice following a period of basal sampling. Clamping glucose with equivalent hyperinsulinaemia required higher GIR in *Cd36*^−/−^ than in WT mice (Fig. 1b–d). *Cd36*^−/−^ mice had higher *R*_d_ (Fig. 1e) and greater per cent suppression of endo*R*_a_ (85% vs 59%) (Fig. 1f). Absolute *R*_a_ approached significance (*p*<0.069) but changes in *R*_a_ and per cent suppression were significantly different. The estimated contribution of *R*_d_ and *R*_a_ to the greater GIR was, respectively, 2:1.

Compared with WT mice, *Cd36*^−/−^ mice had significantly higher *R*_g_ in leg vastus lateralis, soleus, brown adipose tissue and subcutaneous adipose tissue, with the *R*_g_ in heart and gastrocnemius being non-significantly higher (Fig. 1g, h). Overall, the clamps indicate that CD36 deletion improves insulin-stimulated glucose disposal enhanced *R*_d_ and suppressed *R*_a_.

### Muscle insulin signalling

**Fig. 2:**
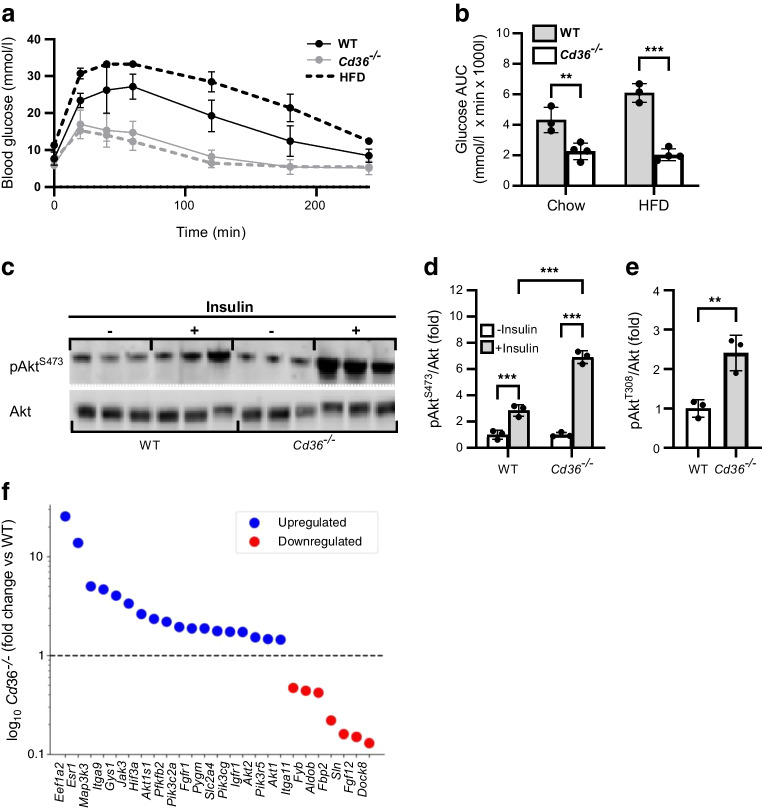
Insulin signalling is enhanced in muscle of *Cd36*^−/−^ mice. (**a**, **b**) Blood glucose levels from IGTT in 5 h-fasted mice fed a chow diet or HFD for 12 weeks (**a**) and the corresponding AUCs (**b**); *n*=3 control mice, *n*=4 *Cd36*^−/−^ mice. (**c**–**e**) Muscle insulin sensitivity. WT mice and *Cd36*^−/−^ mice, *n*=3 per group, were fasted for 5 h, then insulin (7.5 IU/kg i.p.) was administered. The gastrocnemius was harvested 15 min later and probed for CD36, pAkt and total Akt (**c**). Levels of insulin phosphorylated Akt at serine 473 (**d**) and threonine 308 (**e**) were measured, both adjusted for total Akt. Data are shown as means ± SD. (**f**) Altered expression of genes related to insulin signalling and glucose utilisation are shown as fold change of levels in *Cd36*^**−/−**^ mice vs WT control mice (*n*=3 per group). **p<0.01, ***p<0.001

Enhanced glucose tolerance and protection against glucose intolerance induced by high-fat diet (HFD; 60% fat, 7% sucrose) were observed in *Cd36*^−/−^ mice vs WT mice (Fig. 2a, b), confirming earlier findings [13] and ruling out environment- or breeding-related alterations. CD36 enhances insulin phosphorylation of insulin receptor (IR) in human myotubes and CD36 knockdown suppresses insulin signalling and Akt activation [14]. We examined in vivo insulin action on muscle Akt in *Cd36*^−/−^ mice to confirm that it correlates with the clamp data. WT and *Cd36*^−/−^ mice fasted for 5 h were given an i.p. injection of 0.75 U/kg insulin or saline (controls) and the mixed fibre quadriceps harvested 15 min later (see ESM Fig. 1 for CD36 levels). *Cd36*^−/−^ mice had similar basal muscle Akt phosphorylation (S473, T308) to WT mice but insulin-stimulated Akt phosphorylation was enhanced (sevenfold in *Cd36*^−/−^ mice vs threefold in WT) with similar total Akt (Fig. 2c–e). Thus, muscle insulin signalling is enhanced in *Cd36*^−/−^ mice, consistent with the clamp findings.

Gene expression analysis of heart muscle showed upregulation of genes related to glucose uptake/metabolism in *Cd36*^−/−^ mice compared with WT mice (Fig. 2f): *Glut4* (also known as *Slc2a4*) expression was increased 1.8-fold; *Pfkfb*, encoding 2,6-phosphofructo-2-kinase/fructose-2,6-bisphosphatase, expression was increased twofold; *Hif3a*, encoding hypoxia inducible factor (HIF), was increased 2.6-fold; *Gys1*, encoding glycogen synthase, was increased fourfold; *Pygm*, encoding phosphorylase, was increased 1.9-fold; *Pik3c2a* and *Pik3cg*, encoding the insulin mediator phosphatidylinositol 3-kinase (PI3K), were increased 1.7-fold; *Akt2* was increased 1.5-fold), *Akt1* was increased 1.5-fold; the Akt1 substrate 1 gene (*Akt1s1*) was increased twofold; *Igf1r*, encoding primary receptor of insulin like growth factor 1, was increased 1.7-fold and *Fgfr1*, encoding fibroblast growth factor receptor 1, was increased twofold—both are potent stimulators of glucose uptake [31, 32]. Genes encoding integrins, which regulate signalling of PI3K/Akt, growth factors and Src kinases [33] were upregulated three- to fivefold. *Jak3*, encoding Janus kinase 3, which stimulates Akt-independent glucose uptake [34], increased threefold.

### *Cd36*^−/−^ mice have normal transendothelial insulin flux but vascular dysfunction

#### Insulin flux

**Fig. 3:**
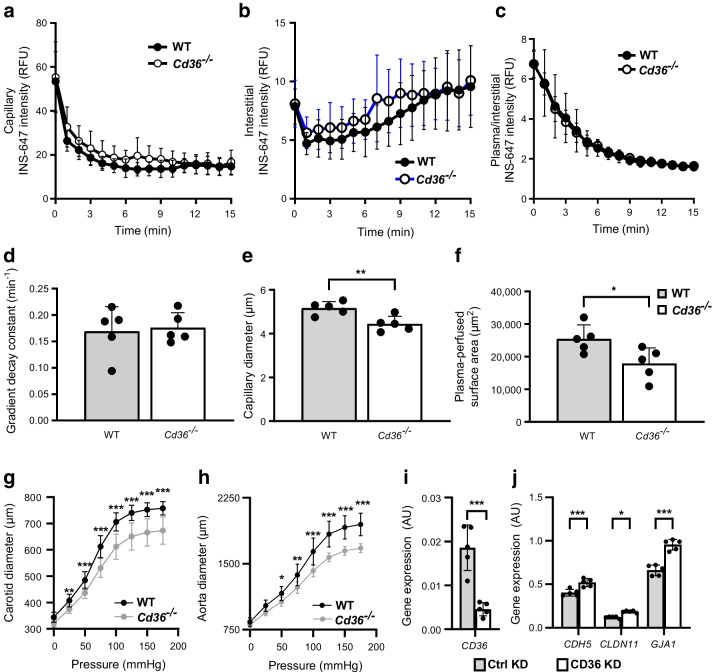
Effect of *Cd36* deletion on transendothelial insulin flux kinetics in skeletal muscle capillaries. A 2 U/kg bolus of insulin-647 was given to WT and *Cd36*^−/−^ mice. (**a**) Capillary plasma INS-647 fluorescence. (**b**) Interstitial INS-647 fluorescence. The interstitial space is defined as the region extending 1–3 µm from the capillary wall. (**c**) Ratio of plasma/interstitial INS-647 intensity. (**d**) The exponential constant of plasma/interstitial INS-647 gradient decay. (**e**) Capillary diameter. (**f**) Perfused capillary surface area. Capillary diameter and perfused surface area were measured using 2 MDa tetramethylrhodamine–dextran fluorescence as a vascular marker. This was further validated using another method (see ESM Fig. 2). (**g**, **h**) Vessel compliance of carotid and aorta as the pressure was increased in steps of 25 mmHg. (**i**, **j**) Expression of junction proteins in hMECs without and with CD36 knockdown. (**i**) *CD36* expression in hMECs treated with control siRNA vs anti-CD36 siRNA.* n*=5 per group. (**j**) CD36 knockdown increased expression of genes encoding junction proteins VE-cadherin 5, claudin 11 and gap junction α-1 as compared with controls. Data are means ± SD. *n*=5 per group. *p<0.05, **p<0.01, ***p<0.001. AU, arbitrary units; KD, knockdown; RFU, relative fluorescence units

Insulin access to muscle cells is regulated by capillaries [18, 19]. We examined whether *Cd36* deletion might affect transcapillary insulin transport using the exposed gastrocnemius and intravital imaging of fluorescent INS-647 as described [23]. Plasma INS-647 dispersion to pericapillary interstitium was unaltered in *Cd36*^*−*/−^ mice (Fig. 3a, b). The decrease in ratio of plasma INS-647/interstitial INS-647, which measures the transfer rate of capillary insulin, was unaffected (Fig. 3c) with gradient decay constants of ~0.15 min^−1^ (Fig. 3d). Thus, transendothelial insulin flux did not contribute to the enhanced insulin-stimulated glucose disposal.

#### Capillary diameter and perfused area

Fluorescence of Rhodamine–dextran was used to determine capillary diameter and the perfused capillary cross sectional surface area. Both were found to be reduced, by ~15 and ~30%, respectively, in *Cd36*^−/−^ vs WT mice (Fig. 3e, f), confirming the muscle data, measurement of renal capillary perfusion using ASL MRI (see ESM Methods for further details) showing a reduction of ~30% (ESM Fig. 2). Immunostaining to visualise heart capillary density showed it was similar in both mice groups (ESM Fig. 3).

#### Vessel compliance

The increase in diameter of the carotid and aorta arteries in response to increasing intravascular pressure showed vessels from *Cd36*^−/−^ mice displayed reduced dilation when compared with those from WT mice, as pressure increased from 0 to 175 mmHg in 25 mmHg steps (Fig. 3g, h). Diminished vessel compliance likely contributed to the reduced capillary diameter and capillary perfused area in *Cd36*^−/−^ mice.

#### Expression of junction proteins

hMECs were treated with control or anti-CD36 siRNA to test the effect of CD36 depletion on junction protein expression. See ESM Methods (Human microvascular cells) for further details and ESM Table 1 for primers. Expression of the abundant VE-cadherin 5 (*CDH5*), claudin 11 (*CLDN11*) and gap junction α1 (*GJA*) modestly increased (20–35%) (Fig. 3i, j). The less abundant claudins 5 and 3 were unchanged while junction adhesion molecule 3 (*JAM3*) was up 20%.

In summary, insulin-stimulated muscle glucose uptake is enhanced in *Cd36*^*−*/−^ mice independent of microvascular adaptations that improve insulin delivery and despite presence of endothelial dysfunction. Microvascular dysfunction normally associates with diminished glucose disposal [35] but in CD36 deficiency it coexists with better insulin-stimulated glucose uptake.

### Individuals with partial CD36 deficiency have impaired microvascular response to insulin but enhanced insulin-stimulated glucose disposal

African American carriers of the G allele of coding SNP 3211938 have 50% of normal CD36 levels [10, 36]. We previously reported that these individuals have endothelial dysfunction [10] but whether this associates with improved or impaired insulin sensitivity is unknown.

#### Characteristics of participants

Table 1 shows the characteristics of the participants. Thirty-five individuals were screened, 14 were excluded or withdrew consent and 21 completed the study. Participants were divided into two groups based on *CD36* rs3211938 genotype. Control individuals (*n*=13) homozygous for the major allele (T/T) had normal CD36 expression, whereas carriers (*n*=8) of minor allele G (G/T) had ∼50% reduced CD36 expression [10, 11, 36]. One participant was homozygous (G/G) with no CD36 expression and was included with the G/T cohort. G allele carriers were 8 years older than non-carriers and their mean weight trended lower. Fasting glucose and triglyceride levels were similar. No participants were hypertensive; systolic BP and diastolic BP were similar. All were healthy and not taking medications except for birth control medication in women.

**Table 1:** Demographic characteristics of study participants (G allele carriers [G/T] non-carriers [T/T])

	G/T (*n*=8; 3 male, 5 female)		T/T (*n*=13; 3 male, 10 female)		*p* value
Characteristic	Mean ± SD	Median (IQR)	Mean ± SD	Median (IQR)	
Age, years	42 ± 7.4	45 (35, 49)	34 ± 7.6	33 (28, 39)	0.019
Weight, kg	79 ± 8.3	82 (69, 84)	89 ± 13.7	87 (80, 95)	0.083
Glucose, mmol/l	4 ± 0.6	4 (4.0, 5.0)	5 ± 1.1	4 (4.3, 4.8)	0.511
Cholesterol, mmol/l	5 ± 1.0	5 (4.1, 5.4)	5 ± 0.6	4 (4.1, 5.0)	0.821
HDL-cholesterol, mmol/l	1 ± 0.4	1 (0.9, 1.6)	1 ± 0.3	1 (1.1, 1.6)	0.567
LDL-cholesterol, mmol/l	3 ± 0.9	3 (2.2, 3.4)	3 ± 0.6	3 (2.3, 3.2)	0.986
TG, mmol/l	1 ± 0.8	1 (0.7, 2.2)	1 ± 0.4	1 (0.7, 2.2)	0.189
SBP, mmHg	124 ± 13.4	130 (120, 128)	120 ± 8.7	120 (113, 128)	0.349
DBP, mmHg	77 ± 10.1	78 (68, 83)	76 ± 9.1	74 (69, 84)	0.857
HR, bpm	78 ± 7.5	69 (64, 102)	68 ± 13.2	66 (56, 79)	0.206

*p* values were calculated with Student’s *t* test

DBP, diastolic BP; HR, heart rate; SBP, systolic BP; TG, triacylglycerol; HDL, HDL-cholesterol; LDL, LDL-cholesterol

#### Insulin fails to increase MBV in G allele carriers

**Fig. 4:**
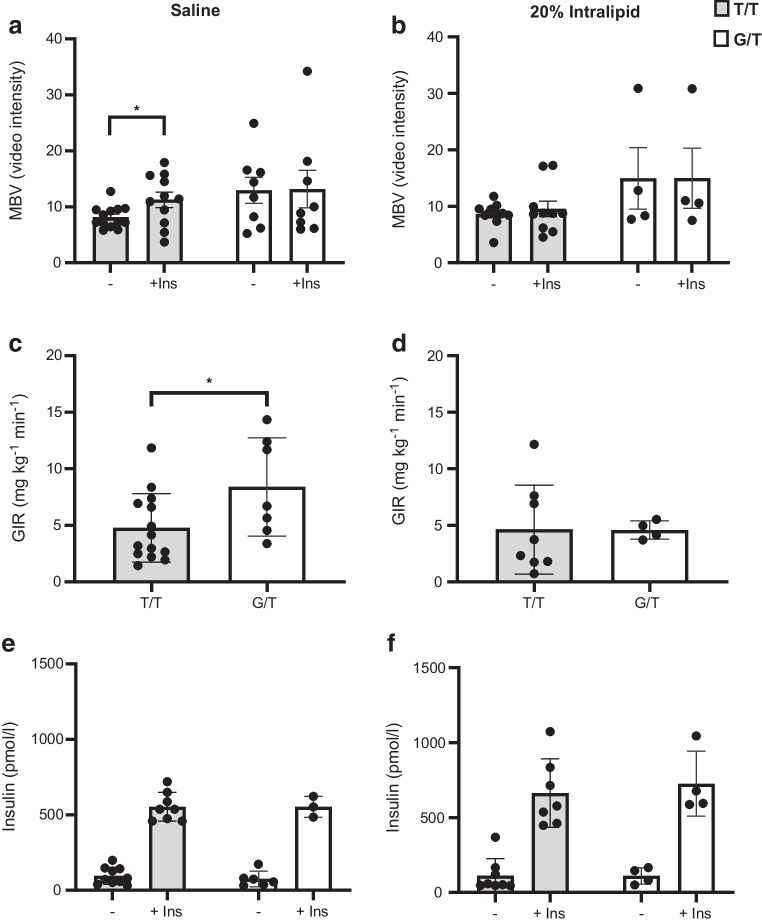
HIEC in individuals carrying the G allele (G/T) of *CD36* rs3211938 with partial CD36 deficiency vs controls non-carriers (T/T). (**a**, **c**, **e**) Participants received a 0.9% (wt/vol.) standard saline infusion (45 ml/h) for 6 h and, during the last 3 h and HIEC. Insulin was infused at a rate of 80 mU m^−2^ min^−1^ for 5 min followed by 40 mU m^−2^ min^−1^ for the remainder of the study. The effect of insulin on the MBV is shown in (**a**). The GIR is shown in (**c**). Circulating insulin levels shown in (**e**) were similar in G allele carriers and non-carriers during basal and clamp periods. (**b**, **d**, **f**) Participants received a 6 h i.v. infusion of 20% Intralipid instead of saline before the 3 h HIEC. The effect of insulin on the MBV is shown in (**b**), the GIR in (**d**). Circulating insulin levels during basal and clamp conditions are shown in (**f**). Data are means ± SD. *p<0.05. Ins, insulin

In non-carriers (T/T), insulin enhanced MBV as video intensity increased from 8.4 ± 0.63 to 11.3 ± 1.37 (35%, *p*=0.05); in contrast, no such increase occurred in G allele carriers (G/T) (Fig. 4a). Infusion of Intralipid instead of saline before the HIEC blunted the effect of insulin on MBV in non-carriers and G-carriers remained unresponsive (Fig. 4b). The velocity of MBF and perfusion (MBV×MBF) (see ESM Methods [Human studies] for further details) were similar for both groups and not significantly altered by insulin or Intralipid (ESM Fig. 4).

#### Enhanced systemic insulin sensitivity in G allele carriers

We next compared the insulin sensitivity of G allele carriers and non-carriers. During the hyperinsulinaemic clamp with a continuous insulin infusion of 40 mU m^−2^ min^−1^, the mean GIR in non-carriers (T/T) was around 5 mg kg^−1^ min^−1^ (Fig. 4c) in line with similar studies [37]. The GIR was 1.9-fold higher in G allele carriers (G/T), suggesting better insulin sensitivity than non-carriers (T/T). Intralipid infusion decreased GIR in both groups, but G allele carriers lost their enhanced GIR as compared with non-carriers (Fig. 4d). Insulin levels during the clamp (Fig. 4e) and the Intralipid infusion (Fig. 4f) were similar for the G/T and T/T groups. Overall, the insulin clamps indicate better insulin-stimulated glucose utilisation in G allele carriers but insulin failure to increase MBV suggests selective microvascular insulin resistance in G allele carriers.

### CD36 depletion in hMEC impairs insulin activation of eNOS

We previously described defective arterial flow-mediated dilation and low cGMP levels in human G allele carriers suggesting reduced NO bioavailability [10]. Endothelial dysfunction with diminished vessel compliance was also documented in *Cd36*^***−/−***^ mice (Fig. 3). Endothelial NO contributes to blood vessel maintenance, and eNOS polymorphisms associate with insulin resistance and type 2 diabetes [38]. We used primary hMEC, which express CD36 and other microvascular signature genes [27, 39] to test role of CD36 in eNOS activation.

#### CD36 depletion of hMECs impairs insulin signalling to Akt and eNOS

**Fig. 5:**
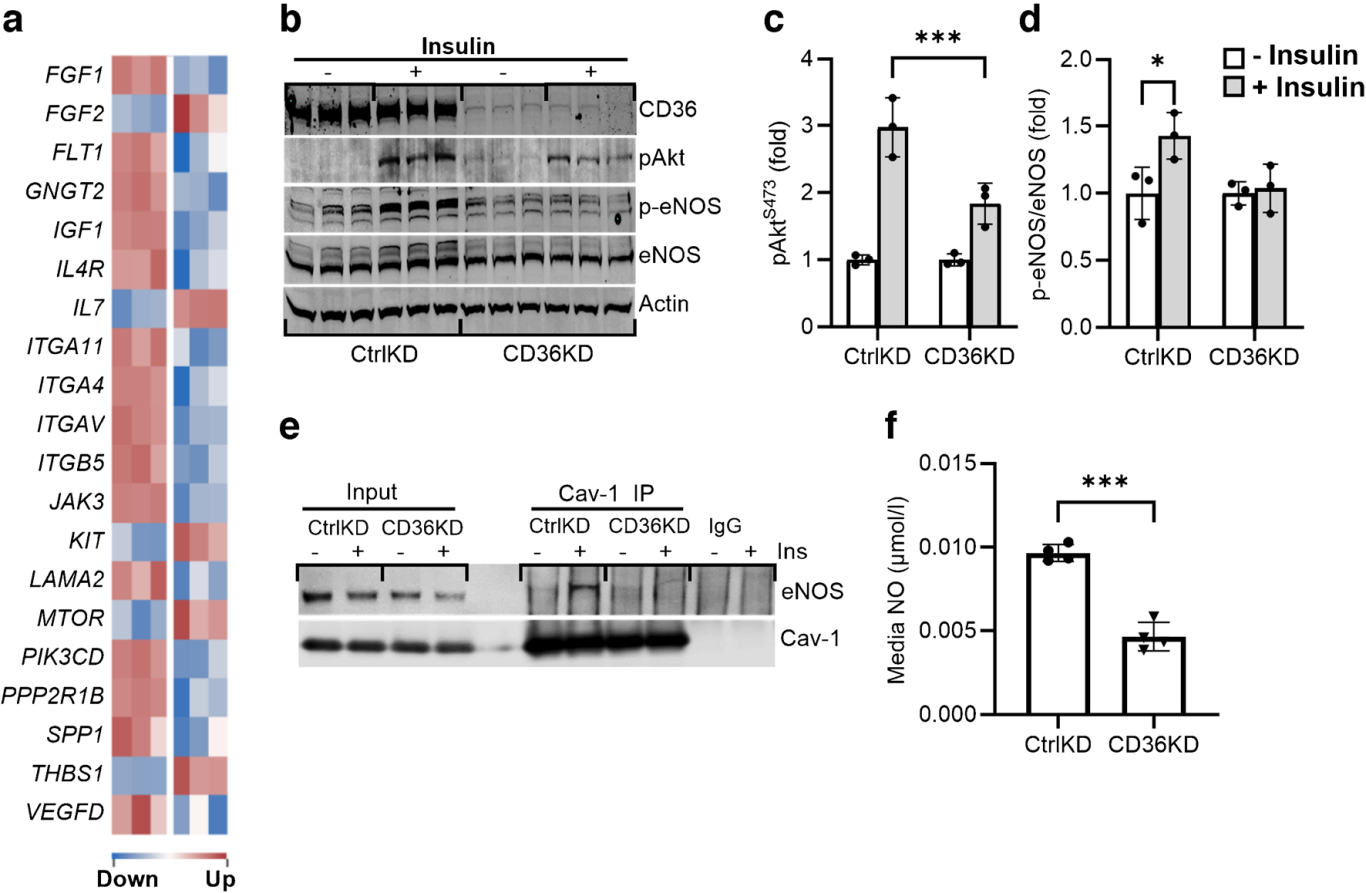
CD36 regulates insulin signalling in primary derived hMEC. (**a**) Alterations in genes related to PI3K–insulin signalling in control hMECs (CtrlKD, hMECs treated with scrambled siRNA) and CD36KD (KD, hMECs treated with anti-CD36 siRNA), *n*=5 per group. (**b**) Western blot of CtrlKD and CD36KD hMECs, showing effect of insulin (100 nmol/l, 5 min) on phosphorylation of Akt and eNOS. (**c**, **d**) Densitometry analysis of pAkt/total Akt and of p-eNOS/total eNOS (*n*=3 *p*<0.05). (**e**) Effect of CD36 knockdown on insulin-dependent interaction of Cav-1 and eNOS. (**f**) Effect of CD36 on regulation of NO production. CtrlKD and CD36KD hMECs were serum starved and subjected to insulin stimulation (100 nmol/l, 5 min). *n*=4 assays. Western blots are representative of three independent experiments. Data are shown as means ± SD. *p<0.05, ***p<0.005. Ins, insulin

CD36-depleted hMECs (using anti-CD36 siRNA) and hMECs treated with control siRNA were subjected to RNAseq to assess alterations in gene expression (Fig. 5a). CD36 depletion increased expression of genes that promote glucose utilisation and cell survival, directly or through activation of PI3K/Akt and growth factor signalling: *FGF1*; *IGF1*; *JAK3*; *PIK3*; and various integrins (*ITG*) involved in PI3K and growth factor signalling [33].

The hMECs tested for insulin responsiveness displayed robust insulin-induced phosphorylation of Akt^S473^ and eNOS^S1177^; both effects were suppressed by CD36 depletion, indicating that CD36 regulates the action of insulin in hMECs (Fig. 5b–d).

#### CD36 depletion suppresses the interaction between eNOS and Cav-1

In endothelial cells, the interaction of Cav-1 with eNOS regulates its internalisation via caveolae, an event linked to its activation [40]. Caveolae internalisation in endothelial cells requires Cav-1 phosphorylation at tyrosine 14 (Y^14^) by the Src kinase. CD36 interacts with Cav-1 and functions in regulating Src signalling. CD36 deletion inhibits Cav-1Y^14^ phosphorylation and caveolae internalisation [27]. Insulin enhances the interaction of Cav-1 with eNOS [41] and Src phosphorylation of Cav-1-Y14 [42]. We speculated that CD36 might be involved in the interaction of Cav-1 with eNOS. IP in control hMECs confirmed that insulin enhances the interaction of Cav-1 with eNOS in these cells [43]; CD36 depletion eliminated this enhancement (Fig. 5e) and reduced insulin-stimulated NO production (Fig. 5f). Thus, CD36 depletion reduces endothelial insulin sensitivity and NO availability. These findings are in line with the failure of insulin to increase MBV in G allele carriers during the clamps (Fig. 4a) and with the previously described defect in arterial flow-mediated dilation and low cGMP levels in these individuals [10]. They are also consistent with the vascular dysfunction (reduced capillary perfusion and vessel compliance) observed in *Cd36*^−/−^ mice (Fig. 3e–g).

Defects in microvascular insulin action and NO production and reduced endothelial compliance are a hallmark of metabolic disease and contribute to the aetiology of type 2 diabetes [44, 45]. Previously, our genome-wide PrediXcan analysis (GWA) of data from Vanderbilt BioVu patient biobank associated low CD36 expression in muscle/heart, and a SNP (rs17236824) close to the transcription start site, with renal, ophthalmic and neurological complications of type 2 diabetes [14]. PrediXcan estimates for GWAS samples Genetically Regulated eXpression (GReX) of a gene in specific tissues and uses GReX to identify genes associated with disease risk [29]. We used PrediXcan again to query whether low expression levels of *CD36* mRNA in blood or arteries associate with type 2 diabetes complications and found strong associations between low vascular CD36 and renal, ophthalmic and neurological manifestations of type 2 diabetes (Table 2).

**Table 2:** Association of genetically determined low endothelial CD36 expression with type 2 diabetes status and complications

Tissue CD36	Effect	*p* value	T2D and complications
Whole blood	−4.323	0.000015	T2D + renal manifestations
Artery, tibial	−4.058	0.00005	T2D + renal manifestations
Artery, tibial	−3.506	0.00045	T2D + ophthalmic manifestations
Artery, tibial	−3.434	0.00059	T2D + neurological manifestations

PrediXcan analysis [29] (see Methods for further details) was applied to 4702 patients of European ancestry and 1484 patients with type 2 diabetes in the BioVU database of Vanderbilt University where health records are tied to a DNA biobank

T2D, type 2 diabetes

## Discussion

Partially CD36-deficient individuals carrying the rs3211938’s G allele have improved GIR, indicating better insulin-stimulated glucose disposal, without the insulin-induced increase in MBV that normally enhances insulin delivery to muscle [18, 19]. Parallel data in *Cd36*^−/−^ mice showed improved GIR with unaltered transcapillary insulin flux, although insulin sensitivity of MBV was not measured in the mice. MBV insulin resistance in CD36-deficient individuals is supported by findings in cultured hMECs where CD36 depletion impairs insulin signalling and eNOS activation (Fig. 5). The suppressed NO generation likely underlies the reductions in vessel compliance and capillary perfusion observed in *Cd36*^−/−^ mice (Fig. 3). It helps explain the diminished flow-mediated arterial dilation of G allele carriers [10] and the failure of insulin to increase MBV in our human G allele carriers (Fig. 4).

Endothelial CD36 delivers circulating fatty acids to muscle and heart, and its deletion reduces tissue fatty acid uptake [15, 27]. It also impairs insulin’s ability to increase MBV for optimising tissue access to glucose and oxygen (Fig. 5). In *Cd36*^−/−^ mice, the muscle and heart adapt to these energy challenges by upregulating glucose utilisation [13] (Fig. 2), which preserves myocyte survival and growth. Muscle expression of genes encoding key glycolytic proteins and primary receptors for the potent stimulators of muscle glucose uptake IGF1 and FGF [31, 32], is upregulated (Fig. 2). Interestingly, CD36-depleted hMECs upregulate the corresponding *FGF1* and *IGF1* (Fig. 5), consistent with endothelial cell modulation of endothelial growth factors as a means of communication with metabolic cells [31, 44]. The decreased capillary perfusion in *Cd36*^−/−^ mice would reduce muscle oxygen supply, which activates HIF-1, -2 and -3, which prime muscle for increased insulin-stimulated glycolytic flux [46]. Hypoxia factor 3α (HIF-3α), the less-studied HIF family member with multiple variants and a gene target of HIF-1 [47], was upregulated 2.6-fold in muscle of *Cd36*^−/−^ mice (Fig. 2), although whether it contributes to muscle’s transcriptional remodelling is unclear. In humans, hypoxia improves glucose homeostasis. Individuals living at high altitude have lower prevalence of impaired fasting glucose and type 2 diabetes compared with those living at low altitude [48]. HIECs show that 10 days of moderate hypoxia significantly increase whole-body insulin sensitivity and glucose uptake in myotubes [49].

The microvasculature could also influence muscle through endothelial cell-released exosomes, the nanoparticles that constitute a major channel for cell-to-cell crosstalk. Fatty acid delivery by EC-CD36 involves generation and secretion of fatty acid exosomes that influence gene expression in human myotubes [27]. CD36 deficiency would alter the exosome cargo (NO, microRNA, transcription factors, fatty acids, phospholipids, ceramides, etc.) and the cargo’s effects in muscle. Finally, altered muscle metabolism could contribute to transcriptional remodelling through changes in signalling metabolites (diacylglycerols, ceramides, acylcarnitines, etc.).

*Cd36*^−/−^ mice are acutely protected from HFD-induced glucose intolerance (Fig. 2). However, Intralipid/heparin infusion in our human studies blunted the greater GIR of G allele carriers and insulin response of MBV in non-carriers (Fig. 4). This lack of protection might reflect 50% (G allele carriers) vs complete CD36 deficiency (*Cd36*^−/−^ mice) but more probably is CD36-independent. CD36 functions in endothelial uptake of fatty acids released from albumin or VLDL but not chylomicrons [50]. Lipid emulsions, like chylomicrons, do not use CD36 [51]. CD36-deficient humans are not protected from hyperlipidaemia, have high circulating fatty acids [10] and chylomicron remnants [12] and are at increased risk of the metabolic syndrome and obesity complications [36].

Association of microvascular dysfunction with enhanced GIR (Fig. 4) contradicts its reported link to insulin resistance [18, 19, 52]. Muscle’s transcriptional remodelling fuels the greater GIR in CD36 deficiency (Fig. 2) and this adaptation might eventually fail in obesity or ageing. Blood flow could become further impaired as CD36 deficiency reduces microvascular repair [53] and has been linked to ischaemic stroke [36]. As illustrated in Table 2 low levels of *CD36* mRNA strongly associate with type 2 diabetes complications, which often involve vascular dysfunction. Targeting NO metabolism in CD36 deficiency could prove beneficial.

Study limitations include the number of participants in the human study, which is relatively small. However, this was mitigated by in-depth metabolic phenotyping before cohort matching and the modest weight difference between cohorts was addressed through adjusting GIR by weight. Similar recruitment criteria were applied to all participants, later separated into cohorts based on *CD36* genotype. Although both self-identified male and female participants were included, the small cohorts do not allow for meaningful sex-based data comparisons. The finding that G allele carriers have a lower weight and better GIR than non-carriers could suggest that CD36 deficiency protects against weight gain and muscle insulin resistance but this needs further testing in larger cohorts. Our participants are African Americans, in whom insulin resistance is relatively more prevalent, so replication in other populations will be important.

## Supplementary Information

Below is the link to the electronic supplementary material.

ESM (PDF 394 KB)

ESM Table 2 (XLS 6785 KB)

## Data Availability

The RNAseq data generated in this study have been deposited in the NCBI Gene Expression Omnibus (www.ncbi.nlm.nih.gov/geo/) under accession code GSE235988 (https://www.ncbi.nlm.nih.gov/geo/query/acc.cgi?acc=GSE235988). Microarray data are available as ESM Table 2. All other data analysed for this study are available from the corresponding author upon request.

## Abbreviations

ASL: Arterial spin labelling
Cav-1: Caveolin-1
CEU: Contrast-enhanced ultrasonography
[^14^C]2DG: ^14^C-labelled 2-deoxyglucose
endo*R*_a_: Endogenous glucose production
eNOS: Endothelial NO synthase
GIR: Glucose infusion rate
HFD: High-fat diet
HIEC: Hyperinsulinaemic–euglycaemic clamp
HIF: Hypoxia inducible factor
hMEC: Human microvascular endothelial cell
INS-647: Alexa Fluor 647-conjugated insulin
IP: Immunoprecipitation
MBF: Microvascular blood flow
MBV: Microvascular blood volume
PI3K: Phosphatidylinositol 3-kinase
*R*_a_: Glucose appearance rate
*R*_d_: Glucose disappearance rate
*R*_g_: Glucose metabolic index (tissue-specific glucose uptake)
SHR: Spontaneously hypertensive rats
WT: Wild-type
