# Supplementary material for: Microvascular insulin resistance with enhanced muscle glucose disposal in CD36 deficiency
Source: Diabetologia. 2024 Nov 6;68(3):662–75. doi: 10.1007/s00125-024-06292-4 (PMC11832635; doi:10.1007/s00125-024-06292-4)
Supplement: Supplementary file 1 — ESM (PDF 394 KB) [file 125_2024_6292_MOESM1_ESM.pdf]

ESM Methods:

Mouse studies:

Transcapillary insulin flux by intravital microscopy: Near-infrared fluorophores were excited by a helium-neon 633nm laser and emitted light detected with a gallium arsenide phosphide semiconductor. For rhodamine-dextran (rho-dex) and near-infrared fluorophores, excitation and emission light were reflected with an MBS 488/561/633 dichroic mirror and imaging used two-channel sequential excitation and detection to avoid bleed-through in an optical section of 8 mm. Imaging region was selected, a background image (t=0) acquired then probe (rho-dex and ins-647) injected through the venous catheter and images acquired every min at t=1-10, at 12.5 and 15min, post probe injection (23).

Capillary perfusion area in kidneys of WT and $Cd36^{-/-}$ mice: Non-invasive Arterial Spin Labeling Magnetic Resonance Imaging (ASL-MRI) was used for a quantitative measure of microvascular tissue perfusion in WT and $Cd36^{-/-}$ mice. Intravascular water is "magnetically labelled" with a non-ionizing radio-frequency pulse and monitored as it transits through the tissue. A flow-sensitive alternating inversion recovery (FAIR)-type ASL-MRI protocol optimized for renal perfusion (57, 58). The data show a 34% deficit in renal perfusion of $Cd36^{-/-}$ mice ($p<0.05$).

Capillary density: Hearts were mounted in OCT medium and frozen in liquid nitrogen. Sections (5µ) were fixed in ice-cold acetone, washed, blocked (Carbo-Free blocking, Vector Labs), then incubated with junctional adhesion molecule 3 (JAM-3) (20ug/ml in PBS) and coverslips mounted using DAPI Hard Set Medium (Vector Labs).

Human studies*:*

Blood glucose and insulin: Plasma was quickly separated from blood collected in chilled EDTA tubes and stored at −80°C. Serum from blood clotted at room temperature (20min) was stored at −80°C. Plasma glucose was assayed by a glucose analyzer (YSI Life Sciences) and serum insulin by multiplex immunoassay (Millipore).

Human microvascular endothelial cells (hMEC):

RNA-seq: The hMEC were treated with control or CD36 siRNA (4392420, assay ID:s2646, ThermoFisher Scientific) and 72 h later total RNA was isolated (TRIzol, ThermoFisher Scientific), cleaned (RNeasy, Qiagen) and submitted to Washington University Genome Technology Access Center (GTAC). Integrated Pathway Analysis (iDEP) web platform was used for normalization and differential gene expression

Expression of junction proteins: hMEC were treated with siRNA and RNA isolated as. cDNA (Superscript VILO, ThermoFisher) was prepared from purified RNA and gene expression quantified using SYBR green

(PowerUp SYBR, Applied Biosystem) on QuantStudio 3 (ThermoFisher). Expression was normalized to the housekeeping gene 36B4.

ESM Table 1: List of primers used for the analyses.

GENE	FORWARD	REVERSE
CLDN3	AAGATCACCATCGTGGCAGG	TCCCGGATAATGGTGTTGGC
CLDN5	TGAAGATTGAGAGCTGCCAGA	ACCCTCTTTGAAGGTTCGGG
CLDN11	GTGACCACCTCCACCAATGA	GCACGTAGCCCGGCAG
JHY	GCGCCAATAACAGGCAACAA	CGTCTCCTGGGAAGGGTAGA
JAM2	AGCAGTAGAGTACCAAGGTGA	AGGTTTTGGCCTTGCTCAGA
JAM3	GTCGTTGCTCGAAATGACCG	TCACTGGCTTCACTTGCACA
CDH5	AGCCCACAGGCACGATCT	ATCTCTCTTTTGGCGCCGGT
GJA1	TGAGTGCCTGAACTTGCCTT	GCCTGGGCACCACTCTTTT
36B4	GTGATGTGCAGCTGATCAAGACT	GATGACCAGCCCAAAGGAGA

ESM Figures:

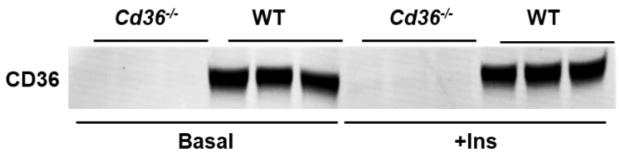

ESM Fig. 1. CD36 levels in quadriceps of WT and *Cd36*$^{-/-}$ mice before and 15 min after injection of 0.75 U/kg insulin.

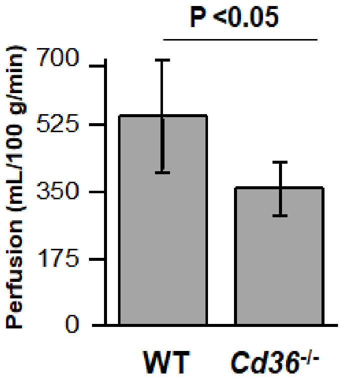

ESM Fig. 2. **Microvascular perfusion in *Cd36*$^{-/-}$ and WT mice.** Renal perfusion was measured using FASL-MRI. Perfusion was 34% lower in *Cd36*$^{-/-}$ mice as compared to WT mice. $p<0.05$, n=5/group.

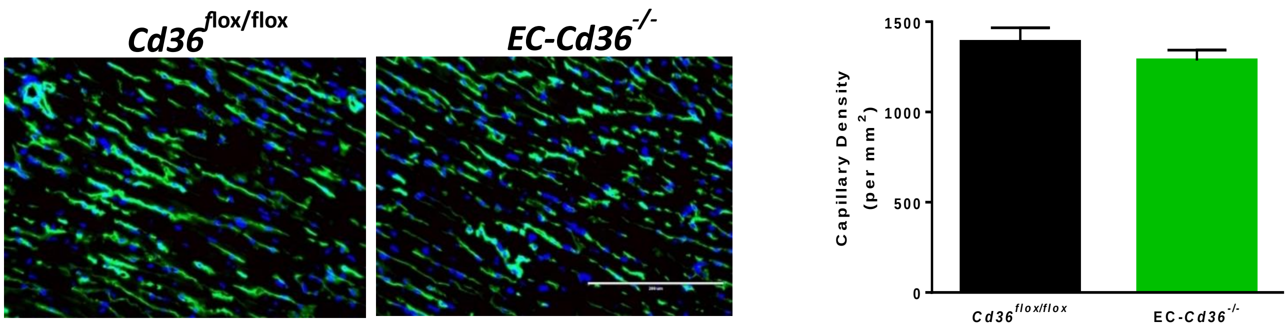

ESM Fig. 3. Heart Capillary Number per mm^2 tissue. Hearts of *Cd36*$^{flox/flox}$ and *EC-Cd36*$^{-/-}$ mice lacking CD36 in endothelial cells were immunostained for junctional adhesion molecule 3 (JAM-3). Images were acquired at 20X from more than 8 fields/sample (2/genotype).

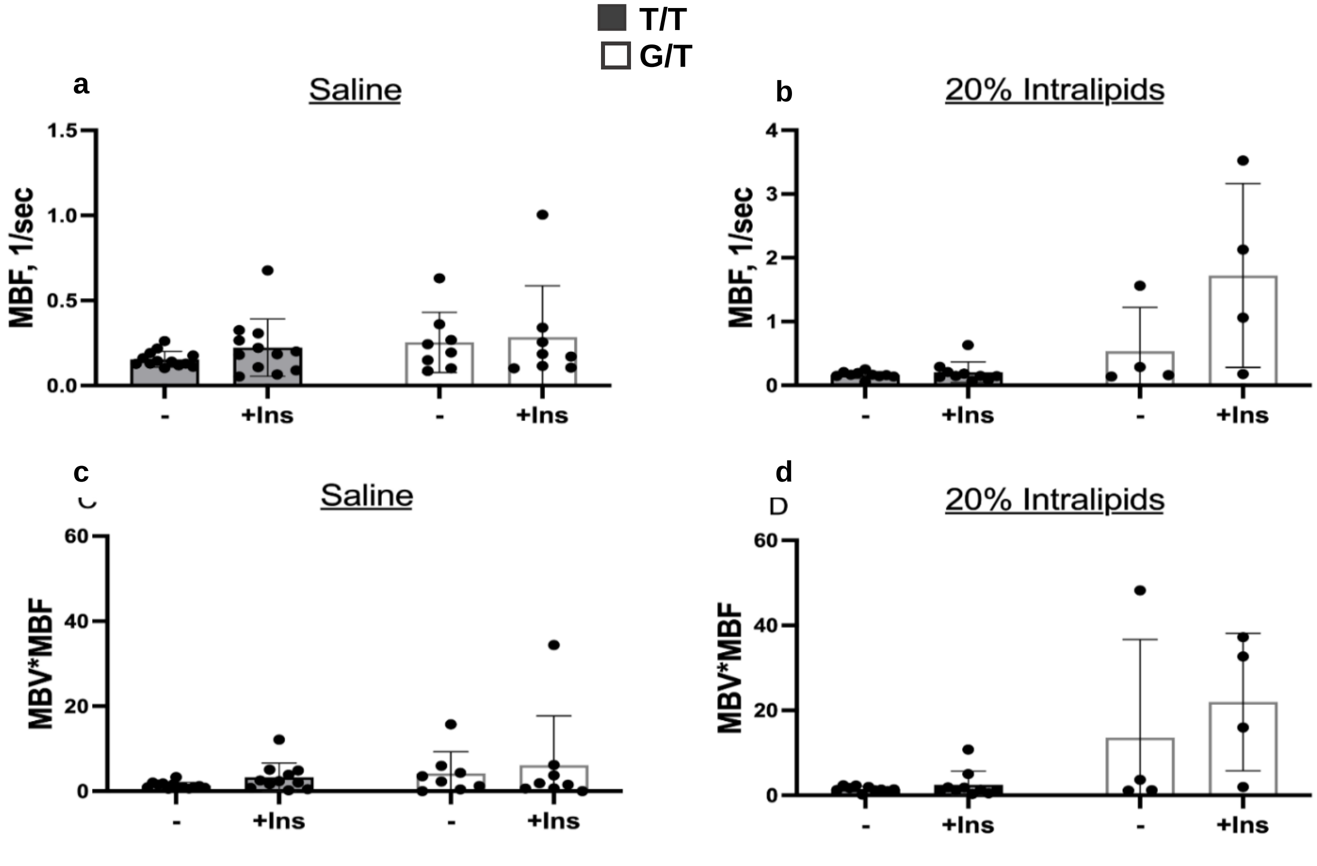

ESM Fig. 4. Velocity of microvascular blood flow (MBF) and overall perfusion (MBF*MVB) in individuals carrying the minor G allele (G/T) of CD36 rs3211938 with partial CD36 deficiency and controls non-carriers (T/T). MBF velocity (**a-b**) and perfusion (MBF*MVB) (**c-d**) are shown before (-) and after insulin (+Ins) for G/T and T/T subjects receiving saline or intralipid before the insulin clamps. Data are means ±SD.
